# Apical Microleakage in Root Canals Containing Broken Rotary Instruments 

**DOI:** 10.22037/iej.v12i3.16656

**Published:** 2017

**Authors:** Mostafa Godiny, Reza Hatam, Atefeh Khavid, Shahryar Khanlari

**Affiliations:** a *Endodontics Department, Dental School, Kermanshah University of Medical Sciences, Kermanshah, Iran;*; b *Endodontics Department, Dental School, Kermanshah University of Medical Sciences, Kermanshah, Iran; *; c *Oral and Maxillofacial Radiology Department, Dental School, Kermanshah University of Medical Sciences, Kermanshah, Iran; *; d *Student Research Committee, Dental School, Kermanshah University of Medical Sciences, Kermanshah, Iran*

**Keywords:** Broken Instrument, Calcium-Enriched Mixture, Gutta-Percha, Leakage, Mineral Trioxide Aggregate, Root Canal Filling Materials

## Abstract

**Introduction:**

Broken instruments in root canals complicate routine endodontic treatment. This study aimed to compare apical microleakage in root canals containing broken rotary instruments filled with mineral trioxide aggregate (MTA), calcium-enriched mixture (CEM) cement, laterally compacted gutta-percha and injected gutta-percha.

**Methods and Materials:**

In this *in vitro*, experimental study, 80 extracted human premolars were decoronated and then the roots were randomly divided into four groups (*n*=20). Root canals were instrumented with Mtwo rotary files. The files were scratched 3 mm from the tip by a high speed handpiece and they were intentionally broken in the apical third of the canals. The middle and coronal thirds of the canals were then filled with MTA, CEM cement, gutta-percha with lateral compaction technique and injected gutta-percha. Apical microleakage was measured using dye penetration method. Data were analyzed using ANOVA and Tukey’s test.

**Results:**

Root canals filled with CEM cement showed the lowest and those filled with injected gutta-percha showed the highest microleakage according to dye penetration depth. No significant difference was noted between the microleakage of CEM cement and MTA or between lateral compaction of gutta-percha and injected gutta-percha (*P*>0.05). However, CEM cement and MTA groups had significantly lower microleakage than laterally compacted and injected gutta-percha groups (*P*<0.05).

**Conclusion:**

Due to their superior sealing ability, MTA and CEM cement are suitable for filling of root canals containing a broken instrument compared to laterally compacted and injected gutta-percha.

## Introduction

Non-surgical endodontic treatment has a high success rate given that adequate cleaning and shaping and efficient obturation of root canals are performed [1]. Efficient obturation must provide a hermetic seal to prevent reentry of microorganisms [2]. An optimal apical seal plays an important role in success of endodontic treatment and health of periapical tissues and can increase the success of endodontic treatment by up to 97% [3, 4]. Absence of apical seal, *aka* apical leakage, has been reported as the most common cause of endodontic treatment failure [5]. 

Root canals can be prepared with hand or rotary files. Rotary files enable faster canal preparation and those made of nickel-titanium (NiTi) can even be used in narrow curved canals due to high flexibility and fracture strength [6, 7]. However, risk of fatigue fracture or breakage due to shear stresses still exists [8, 9]. Canal curvature also serves as a risk factor for file breakage [10, 11]. Thus, despite the attempts of manufacturers, instrument fracture remains a problem in endodontic treatment [12]. Broken instruments complicate adequate cleaning and shaping of root canals and compromise the success of treatment [13-15]. Rotary instruments have a higher risk of fracture compared to stainless steel files [16] and rotary NiTi files have a fracture incidence of 0.4-5% [17]. File fracture mostly occurs in molars and rotary instruments often break in the apical region [18]. Fractured instruments in root canals do not always necessitate surgery or extraction [19]; however, they may compromise healing especially in teeth with periapical radiolucency [20]. Evidence shows that broken instruments remained in the root canal have no adverse effect on prognosis given that the root canals are properly cleaned and sealed [21, 22]. Removal of broken instruments from the root canals is difficult if not impossible and in some cases, the clinician has to bypass the instrument and clean and fill the canal in presence of broken instrument. 

An ideal root canal filling material must have easy handling properties, radiopacity, dimensional stability, insolubility, moisture resistance, sealing ability and biocompatibility [23]. Gutta-percha is the standard root canal filling material commonly used for this purpose. However, it cannot bond to dentin and has poor flexibility [24]. Lateral compaction technique is commonly practiced for root canal obturation with gutta-percha due to relative simplicity and low cost. However, this technique has drawbacks such as risk of void formation and difficult application in curved canals [25]. Injection of thermoplastic gutta-percha was later introduced for better adaptation of gutta-percha to canal walls [25]. This method is fast but is highly technique-sensitive and has a risk of void formation, over-extension or under-filling [26].

Mineral trioxide aggregate (MTA) has been suggested as a root canal filling material due to its optimal sealing ability. Successful use of MTA for apical seal, apical plug and root perforation repair has been reported in many previous studies [27-29]. It is biocompatible and non-toxic and has bactericidal properties [30]. Long setting time, difficult handling, high cost and difficult removal in case of requiring post space preparation or retreatment are among its drawbacks [31]. Calcium-enriched mixture (CEM) cement is another root filling material with hydrophilic and antimicrobial properties. It can provide optimal apical and coronal seal as well [32]. 

Microleakage testing is often performed to assess the quality of root filling using dye penetration method, microbial leakage model, radioisotope tracing or fluid filtration technique [33-35]. Dye penetration technique is amongst the most commonly used methods for this purpose [36].

Considering the existing concerns with regard to management of root canals with a broken instrument, this study aimed to compare apical microleakage in root canals containing broken instrument filled with four different obturation materials/techniques.

## Materials and Methods

This *in vitro*, experimental study was conducted on 80 human premolars extracted for orthodontic or periodontal reasons. The study protocol was approved in the ethics committee of Kermanshah University of Medical Sciences (Grant No.: 3003186).

Sample size was calculated to be 6 in each group considering *α*=95, power of 90% and standard deviation of 0.86 and 4.24 for dye penetration into canals filled with injected gutta-percha and MTA according to a previous study [37]. To ensure reliability of results, 20 teeth were included in each group. The teeth were selected using convenience sampling. 

After collection, the teeth were cleaned and disinfected by immersion in 5.25% sodium hypochlorite for 1 h. They were then stored in 0.9% saline at room temperature until the experiment. The crowns were cut using a diamond bur and high speed handpiece under water irrigation and the roots were divided into four groups for root canal filling with CEM cement (Yektazist Dandan, Tehran, Iran), MTA (OrthoMTA, BioMTA, Seoul, Korea), injected gutta-percha (BeeFill, VDW, Munich, Germany) and gutta-percha (Gapadent, Korea) using lateral compaction technique.

**Figure 1.**
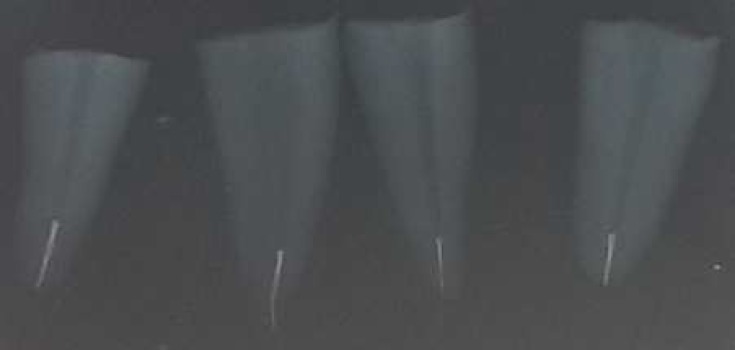
Radiographic image of broken file in the apical part of root canal

**Figure 2.**
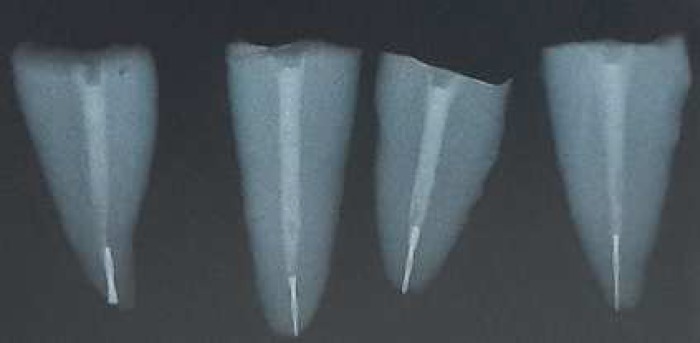
Radiographic image of root canal filling material over the broken file

First, roots were radiographed in buccolingual direction after mounting in acrylic blocks. Working length was determined and the root canals were instrumented with hand K-files (Dentsply Maillefer, Ballaigues, Switzerland) followed by Mtwo rotary files (VDW, Munich, Germany) up to size 25/0.06 to the working length and 30/0.06 to 1.5 mm short of the working length. Recapitulation was performed between files and root canals were irrigated with 5.25% sodium hypochlorite. A final rinse with 1.25% sodium hypochlorite was also performed followed by 17% EDTA and 5 mL of saline. A #30 rotary file was scratched at 3 mm from its tip by a high speed handpiece and was intentionally broken in the canal in the apical region (Figure 1). The middle and coronal sections of the canals were filled with the above-mentioned root canal filling materials/techniques. The roots were radiographed after file fracture and after filling (Figure 2).

**Figure 3:**
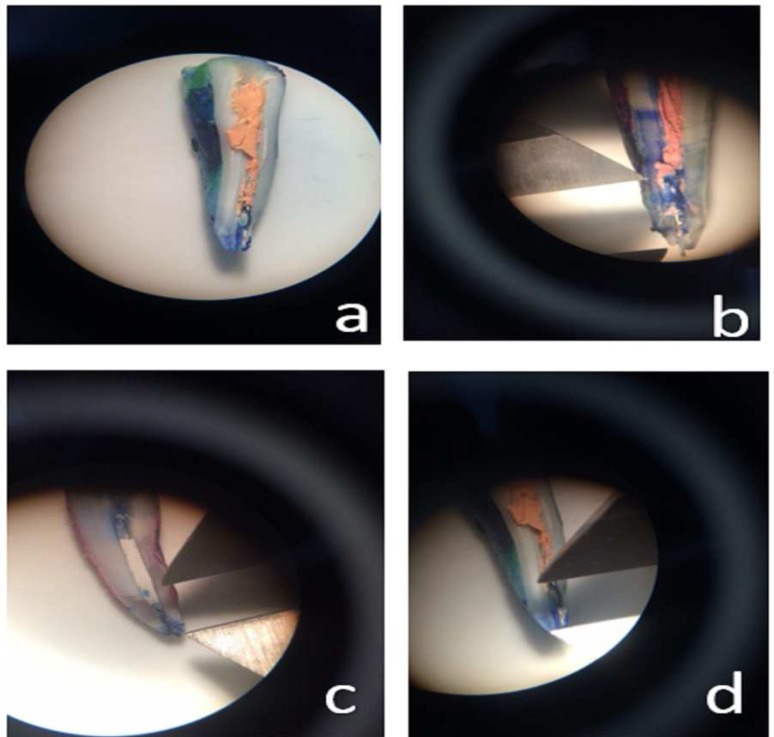
Measurement of dye penetration depth under a stereomicroscope using a digital caliper; A and B) Lateral compaction technique group; C) CEM Cement group; D) Injected gutta-percha group

For assessment of microleakage using dye penetration technique, the roots were coated with nail varnish to 2 mm around the root apex. The coronal orifice was sealed with glass ionomer (GC, Gold Label, GC Corp., Tokyo, Japan). The roots were then immersed in Indian ink for 48 h. The roots were rinsed and mesiodistally sectioned by a cutting saw. The sections were evaluated under a stereomicroscope under ×50 magnification by two observers. Dye penetration depth was measured by a digital caliper (Figure 3). 

Data were analyzed using descriptive and analytical statistics. The mean and standard deviation of dye penetration depth were reported. The Shapiro-Wilk test was used to assess the normal distribution of data. ANOVA was used to compare microleakage among the groups. Tukey’s test was applied for pairwise comparisons. Inter-class correlation coefficient was calculated to assess the agreement between the two observers. All statistical analyses were performed using SPSS software (SPSS version 18, SPSS, Chicago, IL, USA) at 0.05 level of significance.

## Results

**Figure 4:**
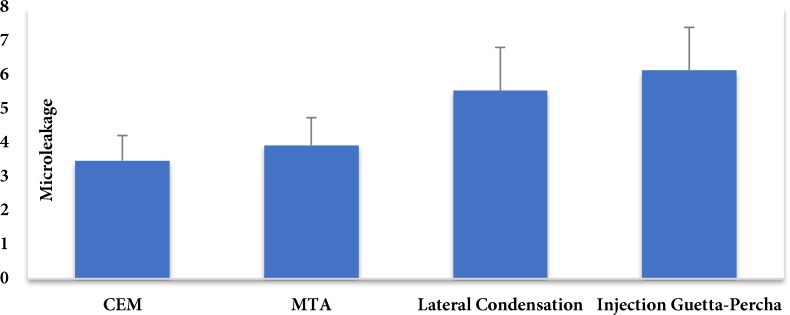
Mean values of dye penetration depth in four groups

The inter-class correlation coefficient was found to be 0.969 between the two observers, which indicated excellent agreement. Microleakage data were found to have normal distribution (*P*>0.05). Table 1 shows the mean and standard deviation of dye penetration depth (indicative of microleakage) and differences in this regard among the four groups. As shown in Table 1 and Figure 4, the mean dye penetration depth was the lowest in CEM cement and the highest in injected gutta-percha group. ANOVA showed a significant difference in microleakage among the four groups (*P*<0.001). Tukey’s test was then applied for pairwise comparisons, which showed no significant difference between CEM cement and MTA or laterally compacted and injected gutta-percha (*P*>0.05) but CEM cement and MTA groups had significant differences with laterally compacted and injected gutta-percha groups (*P*<0.05).

## Discussion

This study aimed to compare the apical microleakage in root canals containing broken rotary instruments filled with MTA, CEM cement, laterally compacted gutta-percha and injected gutta-percha. The results showed that root canals filled with CEM cement showed the least and those filled with injected gutta-percha showed the highest microleakage. No significant difference was noted between CEM cement and MTA or between laterally compacted and injected gutta-percha in microleakage. 

File fracture is a common occurrence in endodontic treatment. The clinicians often attempt to remove the broken instrument but it is not always feasible. Evidence shows that a broken instrument remained in the root canal does not have a significant adverse effect on the quality of root canal seal by filling materials and success of endodontic treatment mainly depends on coronal seal and cleaning of the middle and coronal thirds [21]. However, it has been shown that type of broken instrument also affects the quality of seal provided by restorative materials [38]. Saunders *et al.* [21] showed that microleakage in canals containing a broken instrument was higher than those without it but after filling of root canals with gutta-percha, no significant difference in microleakage was noted. 

Dye penetration technique is a simple and affordable technique for evaluation of microleakage [39]. Several dyes are used for assessment of microleakage such as Indian ink, methylene blue, silver nitrate and Rhodamine B. The pH of dye, chemical reaction and size of molecules affect the dye penetration depth [40]. Indian ink was used in our study since its molecular size is close to that of bacteria [41].

**Table 1:** The mean (SD) of dye penetration depth (μm) (Different superscripted letters indicate significant differences

**Root filling**	**Mean (SD)**
**CEM cement**	3.49 (0.73)^a^
**MTA**	3.94 (0.81)^a^
**Lateral compaction of gutta-percha**	5.55 (1.27)^b^
**Injected gutta-percha**	6.16 (1.25)^b^

We found no significant difference in microleakage between CEM cement and MTA. The same result was obtained by Moradi *et al.* [42] and Kazem *et al.* [36] who compared dye microleakage of different root end filling materials and found no difference between CEM cement and MTA. 

We found no significant difference in microleakage between laterally compacted and injected gutta-percha, which was in line with the results of Altundasar *et al.* [38]. They reported that in canals containing broken instrument, the two techniques had no difference in terms of sealing ability but in absence of broken instrument in canals, injected gutta-percha showed less microleakage. However, Taneja *et al.* [43] reported different results. They reported less microleakage for injected gutta-percha compared to lateral compaction technique in root canals with broken RaCe and ProTaper rotary instruments. Difference in the results of studies may be due to different broken rotary files and different methods of microleakage assessment since Taneja *et al.* [43] used modified glucose penetration technique. 

The quality of seal provided by CEM cement and MTA has also been compared for other applications such as furcal perforation repair [44] and apical seal of resected roots [45] using fluid filtration and bacterial leakage models [46] and no significant difference has been reported; which also supports our findings. CEM cement and MTA are hydrophilic endodontic cements capable of penetrating into small dentinal tubules. Also, they have setting expansion, which results in their better adaptation to canal walls. Moreover, CEM cement forms hydroxyapatite and provides a better seal between dentinal walls and root canal filling material [47]. 

Vizgirda *et al.* [37] compared apical sealing ability of MTA, thermoplastic gutta-percha and laterally compacted gutta-percha and reported superior results for gutta-percha. Difference between their results and ours may be due to different dye penetration techniques used since they used 1% methylene blue. A meta-analysis on laterally compacted gutta-percha and injected gutta-percha reported no significant difference in the quality of apical seal between the two techniques [48], which was in agreement with our results. 

Another treatment modality for a broken instrument in root canal is apical respective surgery and retrograde application of filling material to obtain apical seal. However, isolation of area is difficult and if not well achieved, contamination of the area with blood and fluids may compromise the quality of apical seal. Thus, considering the results of our study, MTA and CEM cement may be used in root canals with a broken rotary instrument. Even if apical surgery is still indicated, only the apical part containing the broken instrument can be resected following root canal filling with these endodontic cements and there would be no need for retrograde filling. Surgical procedure is greatly enhanced as such and more predictable results may be obtained.

## Conclusion

MTA and CEM cement have greater sealing ability compared to laterally compacted and injected gutta-percha and are suitable for filling of root canals containing a broken instrument.
